# The Use of Research for Health Systems Policy Development and Implementation in Mozambique: A Descriptive Study

**DOI:** 10.9745/GHSP-D-21-00694

**Published:** 2022-09-15

**Authors:** Maria Isabel Cambe, Carlos Botão, Janeth Dulá, Elídio Muamine, Sérgio Mahumane, Carla Alberto, Sérgio Chicumbe

**Affiliations:** a Health Systems Program, National Institute of Health, Maputo, Mozambique.; b National Directorate of Surveys and Health Observation, National Institute of Health, Maputo, Mozambique.

## Abstract

Health policy and systems research (HPSR) is generating an increasing amount of evidence in Mozambique, where implementation of a wide range of public health policies and interventions, and innovative technologies and solutions, is underway. We used qualitative methods to explore the relationship between HPSR and policy development and implementation in Mozambique. We conducted a literature review and in-depth interviews with researchers, experts, and policy makers. Through our analysis, we assessed barriers to the use of research evidence in the development and implementation of national health policies and identified potential opportunities to improve evidence use in this context.

We found an increasing number of research institutions producing solid scientific evidence in the country, with activities in health in general and health systems specifically. There is also a growing trend for decision makers and policy makers to use the results of research during the design, formulation, and implementation of health policies. Most HPSR conducted in Mozambique is funded by international donors and focused on research questions of international interest. Therefore, research generated in Mozambique does not always address questions that are relevant to the local health system development agenda.

While Mozambique has a lot of “gray literature” outlets, few of its publications support the translation of research evidence into policy. Much of the evidence generated in country is disseminated through project reports and briefings, not peer-reviewed literature. Furthermore, when the research evidence generated is not locally relevant, results may be published only in English and in scientific articles, instead of in formats useful to Mozambican policy makers—to the detriment of national-level understanding and use. We recommend that research institutions and policy makers in Mozambique collaborate on developing a platform that consolidates HPSR, making it more accessible and useful to policy makers.

## Abstract (precis)

There are still considerable gaps in the process of using research evidence for policy making in Mozambique. We recommend key actions to take to improve the research-to-policy pipeline.

## Teaser

**Key Messages:**

- Despite the large volume of robust evidence available from research conducted in Mozambique, a large know-do gap remains between what the evidence suggests for appropriate action and what is implemented.
- Evidence has neither been disseminated systematically to policy makers nor in a way that policy makers can easily interpret and use, thus it has not been translated into policy priorities in evidence-based policy making processes.
- Current research priorities do not always align with the key policy questions for current health reform efforts because research institutions, influenced by international donor fundings, have focused on implementing research projects that address global research agendas rather than the specific needs of Mozambique.
- Mozambique does not yet have good platforms and mechanisms for researchers to disseminate their findings to policy makers to inform policy questions or research priorities.

**Key Implications:**

- Research institutions, policy makers, and other stakeholders in Mozambique should collaborate to develop platforms that allow for evidence to be accessible and useful to policy makers and set domestically relevant healthy policy and systems research (HPSR) agendas that align with key health policy concerns.
- Domestic and international donors, as well as the Government of Mozambique, should expand funding for research institutions in Mozambique to conduct domestically relevant HPSR.

Resumo em português no final do artigo.

## INTRODUCTION

Linking research evidence to policy development and implementation requires that researchers generate evidence that is useful to policy makers and that policy makers use the evidence generated.^1^ Creating these linkages requires: (1) ensuring that researchers focus their efforts on examining issues and topics that are relevant to key policy questions; and (2) ensuring that policy makers have access to evidence, along with support from researchers to interpret the evidence. In addition, policy decision makers committed to evidence-based policy making must be willing to engage with researchers during complex and political processes that involve several institutions and stages.^2^ Investigating how health policy formulation can be informed by evidence is an important step toward improving the health systems functioning, especially in low- and middle-income countries (LMICs).

In Mozambique, international nongovernmental organizations (NGOs) and national institutions have actively promoted and carried out health policy and systems research (HPSR) since the 1990s. Since then, various aspects of health and health systems in the country have benefited from HPSR-informed innovations. Furthermore, HPSR is generating an increasing amount of scientific evidence in the country—from a wide range of public health, technological, and other innovative interventions designed to overcome urgent health needs. Despite these advances, research institutions in Mozambique continue to have difficulty supporting the translation of evidence into evidence-based public health policy and practice.^2^^,^^3^

Barriers to the use of evidence and its translation into policy are widely recognized in various contexts. For example, doctors are often reported to be reluctant to apply new research findings and evidence-based guidelines to clinical practice.^4^ People who make policy decisions—including administrative managers, legislators, and health policy reformers—reportedly find the use of scientific evidence in developing policy even more challenging.

In general, the most frequently reported barriers to policy makers’ use of research findings include: a lack of skill in interpreting the results and the implications of research; negative beliefs about the usefulness of using evidence; and the tendency for research results to be disseminated in formats that are difficult to read, making it hard for nonexperts to understand. Policy makers’ concerns about the quality of research are also reported to affect whether new evidence is used and translated into policies.^3^^–^^5^

In addition to technical aspects related to the quality and presentation of scientific evidence, research loses its usefulness when it is separated from the environment where it is being applied and the practices of policy makers. One of the most frequently reported barriers is the lack of understanding and nonalignment of the agendas among researchers, policy makers, and decision makers. That is, the problems prioritized for investigation by researchers are not necessarily aligned with the priorities of policy makers. Furthermore, policy designers may not have to contend with the political dynamics encountered by decision makers. As a consequence, when researchers have not addressed policy makers’ and decision makers’ true information needs, their findings are not used by these potential consumers.^5^

When researchers have not addressed policy makers’ and decision makers’ true information needs, their findings are not used by these potential consumers.

The use of evidence-based knowledge is evolving in Mozambique. Many of the barriers to using scientific evidence for the formulation and revision of health policies reported globally are also present in Mozambique. In addition, other factors have been reported, including some related to the context of limited resources.^6^

In Mozambique, there remain barriers to the acceptance of research evidence for formulating health policies and practices, along with a dearth of knowledge about opportunities/mechanisms for overcoming these barriers. The motivation for this study emerged in this context. Our general objectives were to establish which institutions are doing HPSR, document the mechanisms they are developing/using to promote the use of scientific evidence in health policy design and implementation, and identify processes that minimize the gap between the production of scientific evidence and its use by decision makers and policy makers. We present key findings on existing barriers and opportunities to improve the generation and use of research in the health policy-making process and recommendations on next steps to improve the use of scientific evidence for policy making, including additional areas for research.

## METHODS

### Study Design

We sought to explore the relationship between HPSR and health policy development and implementation in Mozambique. In preparation, we initially reviewed the available evidence on Mozambique in the literature. We focused on evidence-based policies, asking: What drives policy formulation? What influences the use of evidence in the policy-making process? What is the importance of evidence-based policy making? And how is research evidence used in decision making? Based on our review of the literature, we designed a cross-sectional qualitative study that incorporated an earlier mapping of Mozambique’s public and private HPSR institutions and key informant interviews with specialists from these institutions. All authors had rotating and multipurpose roles throughout the study. We took turns serving as interviewers and annotators during the interviews, and we were all involved in analyzing the data collected and preparing this article.

### Study Participants and Sampling

For our study, we sought out key informants in 2 categories. The first category was health policy and systems researchers with extensive experience conducting research in health systems at national and subnational levels. This group included the senior managers or researchers who had either the greatest number of years or at least 5 years of experience in the organization. Also included in this category were representatives of domestic, private, or public institutions or organizations that produce HPSR and have had an active HPSR profile during the previous 3 years. In the second category, policy makers, our interviewees represented national directorates and other government departments, the legislature, or other organizations responsible for making health policies at the national and subnational levels. This group included members of Parliament, the Ministry of Health, other high-level national health officials, health secretaries, and local chief executives.

We selected interviewees via convenience sampling, based on previous policy research mapping activities, and purposive selection of representatives of institutions known to implement policy research or HPSR, as well as legislators and policy decision makers.

### Data Collection

We interviewed 32 key informants throughout September and October 2020, predominantly in the capital, Maputo City. Some participants from other provinces were also included. Given the limitations imposed by the COVID-19 pandemic and interviewees’ preferences, we administered some interviews in person and others via the Zoom and Skype platforms. Our interview guides included semistructured and open-ended questions related to the use of research for policy development; the guides were adapted depending on the type of institution and role of the interviewee. The interviews were conducted in Portuguese. All interviews were audio-recorded and fully transcribed.

### Data Analysis and Management

We collected, recorded, transcribed, and analyzed all interview data through thematic analysis. We applied predefined themes and codes, based on the literature review, to the transcriptions. While thematic analysis was being carried out, additional codes emerged within the themes and were incorporated. We added these deductive codes to a thematic structure matrix used to identify the broader narratives. Microsoft Excel was used to manage and filter data, quantify occurrences of emerging and predefined themes, and aggregate the coded data.

### Ethical Considerations

The study was approved by the Institutional Review Board of the National Institute of Health of Mozambique (089/CIBS-INS/2020). All recommended and required ethical procedures for carrying out scientific research in Mozambique were followed. Interviews were conducted only after free and informed consent was obtained from study participants.

## RESULTS

### Overview of the Interviewees and Data

We interviewed 32 individuals (Table), of whom 56% were men and 43% women. Regarding the legal frameworks of the 32 institutions represented, 42% were governmental, 52% non-governmental, and 6% academic.

**TABLE.** Characteristics of Policy Makers and Health Policy and Systems Researchers Interviewed, Mozambique

	No. (%), N=32
Gender	
Female	14 (44)
Male	18 (56)
Nationality	
Mozambican	31 (97)
Other	1 (3)
Type of institution, by legal framework	
Academic	2 (6)
Governmental	14 (42)
International nongovernmental	8 (26)
National nongovernmental	8 (26)
Age group, years	
30–35	4 (14)
35–40	5 (16)
40–45	5 (16)
45–50	5 (16)
50–55	1 (3)
50–55	5 (16)
55–60	5 (16)
65–70	1 (3)

According to most respondents, the evidence produced in Mozambique is of good quality but not always used for decision making. Whether or not evidence is used depends on several factors that relate to both the research and its intended audience. These include the need for and urgency of the research, the research objectives, the type of research results, how results are disseminated, the information that the available evidence provides, the profile of the decision maker, the urgency with which the subject must be debated and resolved, the level of perception around the context of shared evidence, the context in which the evidence should be used, and the relevance of the subject under debate from the point of view of the decision maker.

From our interviews with policy makers and researchers, several topics emerged: the evolution of scientific evidence production in Mozambique, factors that contribute to the use (or not) of scientific evidence to influence policies, how research institutions influence health policies, and how institutions define their research priorities.

### Evolution of Research Production in Mozambique

Promoting research on health policies and systems, as well as other health-related topics, has been encouraged and carried out since the 1990s, with mixed results in LMICs including Mozambique. We performed a basic analysis—based on interviews and a previous mapping exercise—of the progress of research production in Mozambique by tracking the number of research institutions in the country and the cumulative scientific production between 1990 and 2020 (Figure).

**FIGURE.**
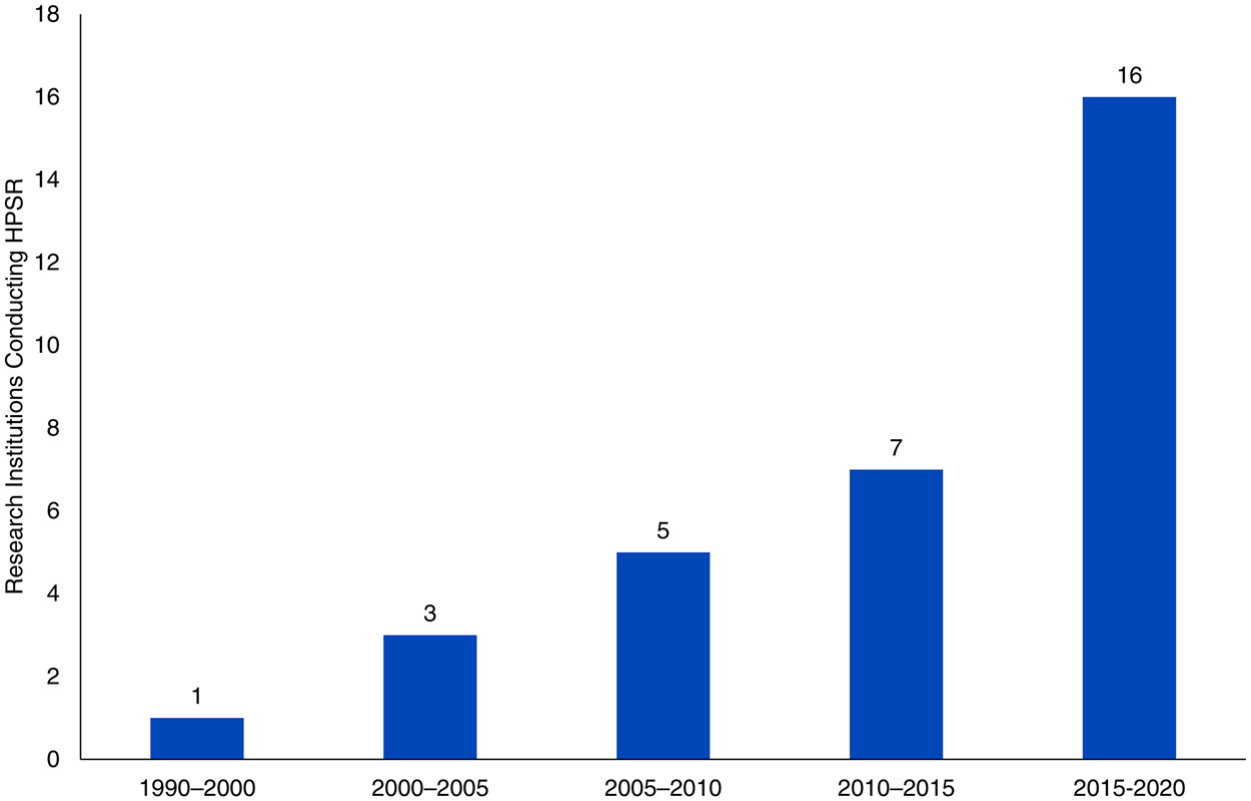
Number of Research Institutions Conducting HPSR in Mozambique Abbreviation: HPSR, health policy systems research.

In general, we found that since the 1990s, institutions in Mozambique have been conducting HPSR activities to address both the country’s needs and the priorities of international funders. These include HIV and AIDS, TB, malaria, and childhood immunizations. Researchers, as well as government and nongovernmental institutions working in these areas, have been gaining experience and raising the quality of research, while narrowing their areas of focus.^7^^,^^8^

The volume of scientific production in Mozambique, combined with the viewpoints of the majority of interviewees, suggest that research on health in general and on health systems in particular has generated robust evidence. However, the evidence frequently has not been disseminated systematically and comprehensively, nor has it been directed to the appropriate audiences. Further, often this evidence is not translated into indicators for health policies or programs. These findings represent a gap in the capacity of institutions and researchers to influence policy makers and health policies.

### Factors That Contribute to the Use of Scientific Evidence to Influence Policies

#### Mechanisms to Dissemination Research

The way in which research evidence is disseminated represents one barrier. Few Mozambican researchers have published their results in scientific articles, indexed journals, or policy dialogues. Thus, the findings are not accessible to either the global scientific community or the public for use in policy design. Data and key findings are typically presented in long, detailed reports, a format that has little appeal for political decision makers. According to interviewees, research results are generally disseminated as either physical or digital reports through the Ministry of Health (MISAU) Portal, via television or radio spots, during presentations to institutional boards of directors, during multisectoral technical working groups, on open research days, on health days, and in scientific sessions. Mozambican research institutions—such as the medical school of the University Eduardo Mondlane, National Institute of Health, and Manhiça Health Research Center (CISM)—organize events to present their recent research results to representatives from various government institutions, international and national NGOs, and other interested parties.

Furthermore, interviewees mentioned instances when important research results were not disclosed or made public at all. No common repository or clearinghouse for health sector research exists in Mozambique. Thus, research-based evidence is not routinely made available for policy makers to use in their deliberations. Instead, dissemination to policy makers and the public is done by research institutions differently in each case. This is a significant limitation to using evidence to generate health policies.

Research-based evidence is not routinely made available for policy makers to use in their deliberations.

Interviewees also noted that researchers could better inform policy makers throughout the research process, not just at the end. They suggested that researchers could even include policy makers in their process. For example, a consultative process with appropriate platforms in place could allow policy makers and researchers to share their interests and findings (respectively), strengthen the relationships/collaborations between them, and facilitate dialogue and consensus.

#### Emphasis on Political and Global Priorities Over Evidence-Based Policies

Interviewees perceived most research conducted in Mozambique to be credible, based on their understanding that it was conducted in a complex way. However, they were unanimous in suggesting that political relevance is prioritized over evidence-based practices, in the context of both designing policies and implementing research projects. Contributions from or consultations with research institutions and researchers are not regularly or systematically considered by decision makers and policy makers.

*When I decided to build the hospital … I didn’t need scientific evidence, but I knew, had a plan, and I wanted to build the infrastructure, which could be well equipped … At that time, I had negotiated with the donor, and they had the money to do. So, it is why we have the hospital and the Health Training Institute in the same place*. —Political decision maker

Interviewees also observed that the perception, sometimes backed by experience, that most HPSR institutions respond to indicators established by global funding agencies to address global policy commitments such as the Sustainable Development Goals. Although HPSR evidence is produced with the intent to influence policies in Mozambique, most of it does not respond to the specific needs of the country. This disconnect has contributed to health policies not being informed by local evidence.

Respondents noted the perception that most HPSR institutions respond to indicators established by global funding agencies to address global policy commitments.

*To answer relevant political questions currently for the HIV area here in Mozambique, all indicators are designed to accommodate the experiences and needs of global interests. Policies are set and designed far outside the technical capabilities of the National Health System (SNS) and this causes a disparity between the global commitment and local needs and commitments since these indicators were designed based on evidence that does not fit the capabilities of the SNS.* —Political decision maker

### Research Institutions’ Influence on Health Policies

Interviewees acknowledged research institutions’ privileged proximity to policy makers and constant collaboration with the government, civil society, and national and international financing agencies. They saw the acceptability and influence of these institutions as derived from their role in providing consultation and guidance on a range of important public health issues, such as malaria (assisting in planning the distribution of mosquito nets); immunization (assisting in micro-level planning and management of vaccination campaigns); and HIV (developing messages and strategies for behavior change and distribute condoms and lubricating gel for prevention). Because of the relevance of their research and the interventions they manage, these institutions have prestige and recognition from the government and other partners.

Interviewees reported that research institutions are able to exert influence on political decision makers because of the regular contacts and collaborations between them.

*We, in terms of positioning and political influence, are on a good level, and as a result, we were invited to be part of the technical group.* —Political decision maker

*Foundation for Community Development (FDC) is an institution seen as mitigating HIV within the community. Through the first project, we created links not only with National Combat Council AIDS (CNCS) but also with Ministry of Health (MISAU), which helped to leverage many projects. Later, we joined the health environment project, having identified three provinces … through the extended vaccination program (PAV).* —Research institution representative

Interviewees reported that research institutions are able to exert influence on political decision makers because they are in regular contact.

Although research institutions collaborate in these programs in part to explicitly influence policy, interviewees felt that this collaboration still represents a challenge for the institutions. They reported that evidence and researchers’ recommendations are not always considered due to policy makers’ difficulties in interpreting evidence and their need to make quick decisions.

Interviewees were asked about the relationships between research institutions and MISAU, as most respondents were MISAU “collaborating partners.” Most respondents described the relationships as healthy and continuous. However, some research institution representatives said the quality of their research may be threatened by difficulties accessing information and data from government institutions. These difficulties were reported as generally related to bureaucratic procedures, coupled with the fact that the procedures for technical and ethical review and approval are time consuming. Instead of going through such lengthy processes, some institutions reportedly choose to carry out urgent studies without due approval from the Ministry’s Bioethics Committee. Some interviewees also claimed that bureaucratic constraints limit access to evidence when it is needed. According to them, these instances are outliers, but their endeavors may negatively affect policy makers’ perceptions of research quality and relevance. Improving and streamlining formal mechanisms for research institutions to collaborate with MISAU could improve both the quality of research conducted and the subsequent use of research evidence for decision making by the government.

### Research Institutions Determine Competing Research Priorities

According to most respondents, 2 main scenarios determine how HPSR agendas are established in Mozambique. In the first scenario, research projects are designed and executed in line with a global agenda. According to participants, in these cases, global agendas and financing provided by international entities dictate the direction and type of research done, including defining the indicators that must be addressed. Doing research in the health area, according to interviewees, is expensive; a perception exists that institutions that do not align with the international agenda may lose funding opportunities. Institutions fear that without international donor funds for research, they would lose opportunities to conduct work, be recognized, and have credibility. Ultimately, research institutions in Mozambique perceive that lack of international funding threatens their survival.

*It is not difficult to know how to set a priority because the world has an agenda, the funders have an agenda, and if you leave the agenda you risk having difficulty in obtaining funding and being heard … Attention to global needs and regional needs must be managed. But it remains to be seen where the country wants to go.* —Research institution representative

According to most participants, research institutions in Mozambique typically access funding by responding to calls for grant proposals issued by donors. Most of the funding agencies are international, such as the Global Fund, development banks (including the World Bank and the African Development Bank), the World Health Organization, and the United States Agency for International Development.

In the second scenario, research priorities are identified based on the national Social Economic Plan,^9^ which addresses activities and indicators from the government’s Five-Year Plan.^10^ In this scenario, researchers are focusing their efforts on examining nationally and locally determined health indicators.

*The government's Five-Year Plan is a document that reflects what the government has committed to and is approved by the Assembly of the Republic*. —Political decision maker

However, the government does not provide significant funding for research in line with the plan. A domestic source of funds is the National Research Fund for Investigation (Fundo Nacional de Investigação, or FNI), which falls under Mozambique’s Ministry of Science and Technology. However, FNI does not finance research of great magnitude, such as large surveys or clinical trials.

Thus, the more viable scenario, from the point of view of research institutions concerned with their financial sustainability, is the first one. As a result, their research priorities may be defined according to the availability of funding rather than nationally determined goals.

Several research institutions are involved in HPSR and are perceived as collaborating with the government and complementing each other while using different approaches.

*The performance of the health sector is sensitive due to the large number of partners involved, and also the mechanisms used. However, sometimes there have been discussion forums where institutions establish partnerships to act in a determined area of health.* —Decision maker

It should be noted that the current context of design and implementation of research in health policies and systems was described as evolutionary and robust given the existence of several research institutions, which collaborate with and complement each other, through different approaches, using the simplest themes and methods to the most complex themes and methods. Respondents perceived the profiles of the institutions and mechanisms used to influence policies as closely linked with the sources and availability of research funding. Most HPSR institutions are funded by organizations that also support programmatic health activities, according to respondents. However, research institutions face problems related to the complete lack or insufficiency of funds to finance their research. Limited available funding for research affects all of Mozambique’s research institutions, not only those in the health arena, constituting an important challenge for the country’s generation and use of scientific evidence.

### Policy Decision-Making Process

According to both the researchers and the policy makers interviewed, all policies in Mozambique are informed, explicitly or implicitly, by evidence, despite the difficulties previously described. They said that policy makers always make evidence-based policy decisions, but that the evidence may not be from Mozambique. Many interviewees reported that the demand for evidence for health policy development and implementation has increased over the years. However, they stated that procedures and platforms to encourage the generation and use of scientific evidence in designing health policies are still scarce. They also recognized that projects are implemented without the use of evidence but stated that both researchers and policy makers are aware that research is necessary to produce evidence to formulate strategic policies and programs.

*Research, and institutions that design research, in health systems have expanded greatly. There has been huge investment in human resources, institutional training, and others. For example, in human resources, there are no policies that were developed without evidence. All policies we design have some basis and “some basis” means evidence. Without any evidence, in one way or another, when you want to implement an action, strategic or political, even if it is not explicit about it, you are using evidence.* —Policy maker

*Policy- and decision-making processes take place in a complex social contexts. These may be influenced by: technology (or lack thereof), cultural norms (that can restrict access to health services), geographical considerations, and institutional limitations.* —Research institution representative

Platforms to encourage the generation and use of evidence in designing health policies are still scarce.

Government policy requires joint action among different disciplines. These factors combine to determine whether a given policy decision is appropriate for the territory where it will be implemented. Understanding all these factors requires evidence. The team of people involved in policy decision making must also have multiple perspectives. In the case of health policies, the team should include people with expertise in public health, medicine, demography, statistics, and public policy, among others.

Concerning the dissemination of research, the government is frequently the target audience for research-based evidence presented in reports and policy briefs, or at conferences, scientific open days, or formal meetings. As one decision maker noted, most research institutions have focal points from the relevant ministries involved in their working groups; such participation facilitates the dissemination of results to the relevant ministries. However, regardless of which formats are used, the question remains: is the available evidence translated into policy priorities through the use of relevant indicators? Based on our analysis, and according to the respondents, this step still constitutes a major gap in the decision-making process in Mozambique.

## DISCUSSION

We are among the first to explore the use of HPSR in Mozambique, including the relationship between the generation of scientific evidence and the process of formulating health policy. To develop a picture of the current situation, we sought to understand the viewpoints of producers of scientific evidence and those of policy makers and implementers. We also sought to identify opportunities to improve the use of research results in the development of health policies.

We found that Mozambican researchers and policy makers are committed in theory to the implementation of evidence-based policy; they perceive it as a way to improve the quality of care provided to citizens. Indeed, the study found that research is already being used for the creation of evidence-based health systems policies in Mozambique, and this use appears to be increasing. The use of research for design and implementation of health policies and systems strengthening has been described in our interviews as both “evolutionary” and “robust.”

However, implementing the use of evidence in policy making is an inherently complex process that entails interactions among many different stakeholders (including individuals and institutions, health care providers and policy makers, researchers and decision makers), each with their own objectives and concerns. These dialogues would allow various stakeholders to share research-related benefits more effectively.

Implementing the use of evidence in policy making is an inherently complex process that entails interactions among many different stakeholders.

The application of knowledge produced by scientific research, the availability of updated and reliable information, and the observance of the values of interested people and existing legal frameworks are not yet widely disseminated ideas to make health services and systems more effective and sustainable. In Mozambique, there is a Quality Scientific Evidence-Based Policy Network, but its work does not align with global efforts to connect and strengthen research evidence to actions in health systems.

The process also faces many obstacles, particularly related to the creation and availability of information directly relevant to areas of immediate interest to policy makers. Research institutions have difficulties in translating their findings into policy recommendations. According to our data, there is also a lack of knowledge and skills among policy makers about how to access evidence (from, for example, journals or databases), and how to select, interpret, and apply HPSR results. To ensure that research-to-policy dialogues occur and that evidence is used to define policies, all interested stakeholders should commit and be open to collaboration. The process of establishing platforms for sharing information and policy dialogue will require careful management and clarity on procedures.

As other studies in the current literature indicate, to design effective evidence-based public health interventions, the best available evidence must be used to make decisions about the provision of health services to communities and populations.^11^^,^^12^ Our findings agree with this notion.

Research institutions often develop their research agendas based on the funding available, rather than national policy priorities. Health policy makers and implementers in the country acknowledge the importance of evidence-based practice but do not have a robust culture of using locally generated research evidence to make decisions. This gap exists for several reasons, but perhaps the most prominent is that conducting robust research requires significant time and technical expertise to capture and analyze evidence. These requirements do not always harmonize with the urgent nature of political decision making. There are likely other misalignments among the agendas of researchers, policy makers, and decision makers. Furthermore, Mozambique lacks a well-organized public repository for research findings. The existing mechanisms used to disseminate research results generally have restricted audiences, making it difficult for politicians and different interests to access and use evidence produced through research conducted in the country.

Further, research institutions in any given country should conduct research that accounts for and addresses the country's sociopolitical situation, including reviewing previous policy impacts, to provide policy makers with appropriate evidence to improve existing policies and develop new programs. Evidence generated through responsibly conducted research can provide important and useful inputs to assist in decision making. Using evidence strengthens decision making by adding scientific weight to the experience of the decision makers.^8^^,^^12^

Some authors have noted that, despite the growing production of scientific evidence for public health decision making globally, a great gap remains between the appropriate courses of action suggested by scientific evidence and what is actually implemented (the so-called “know-do gap”).^7^ We found this gap in Mozambique: political decisions are often made without reference to, and not in line with, evidence from research.^7^^,^^13^

In Mozambique, a great gap remains between the appropriate courses of action suggested by scientific evidence and what is actually implemented.

We found this gap in Mozambique: political decisions are often made without reference to, and not in line with, evidence from research.

One way to overcome this gap in Mozambique may be to create a platform (or regular meetings) for the translation of knowledge through clear communication on and interpretation of research findings. This exchange would provide a foundation for regular interactions among researchers and decision makers. Ongoing dialogue would enable collaborations between researchers and policy decision makers as they synthesize, disseminate, discuss, and ethically apply knowledge created through research.

In high-income countries, additional barriers to bridging the know-do gap have been identified. For example, despite public health managers participating in workshops and professional councils or having local surveillance data to inform their decision making, reports suggest their difficulties in using scientific research results persist. This issue is reportedly caused by the lack of support and skills in interpreting research, and it results in the lack of policies based on evidence and even inconsistent policies.^7^

In LMICs, the know-do gap is even more extensive, as socioeconomic, political, and other factors limit resource availability for designing and implementing health policies based on evidence.^9^ We found that the type of funding available conditions the type of research and results achieved. According to our findings, most research designed and implemented in Mozambique responds first to the needs of and indicators established by the global and international funding agencies.

Understanding the effects of public health policies is central to improving population health. Given the dynamic nature of policy and the limitations of research-based evidence, we can rarely prescribe exactly the “right” design for a health system. However, research can be an invaluable tool for projecting probable outcomes, thereby setting realistic expectations of likely advantages and shortcomings of various policy options.

The technical elements of robust research can sometimes act as barriers to its use. The time required to capture and analyze evidence may not align with the urgent nature of political decision making. Further, one of the most commonly reported barriers to the use of evidence is the misalignment of agendas among researchers, policy makers, and decision makers. That is, the problems researchers are interested in do not always align with the priorities of policy makers. In these cases, the intended consumers of HPSR may not use available evidence because researchers have ignored either their time limitations or their priority issues.

Evidence-based policy making does not restrict innovation. When the evidence is unreliable, uninformative, or simply nonexistent—or when previous policies have clearly failed—it is essential to explore new ways of achieving policy objectives and, possibly, pilot new policies. In such instances, it is important for researchers to assist policy makers as they assess these efforts to generate evidence for future political decisions.^9^

### Limitations

The design and implementation of policies involves a long process of complex interactions among various actors at different levels, each with different interests and approaches. Given the observational and cross-sectional nature of this study, the data generated did not allow extensive inference or extrapolation on these dynamics. Further, given that this study is one of the first of its kind in Mozambique, its representativeness cannot be assessed.

## CONCLUSION

Rigorously conducted HPSR should support policy makers as they experiment with innovation and make informed decisions, with the goal of formulating and implementing policies that contribute to improved health outcomes and health system functioning.

Despite the increasing quality and quantity of scientific research conducted in Mozambique, considerable gaps remain between the evidence produced and its translation into evidence-informed policy. We conclude that the impact of research results in the formulation of policies and decision making is currently at an incipient stage in Mozambique.

To enhance our understanding of the challenges, we recommend further research into recent instances of developing and implementing key HPSR agendas. This analysis would enhance our understanding of the various stages and processes that take place, thereby allowing us to identify opportunities to introduce more use of research at health system key points of this research to improve policies. We have created a baseline for further reflection and evaluation of existing policies and decisions. Our findings also offer some guidance on the use of evidence in the construction of new health policies. Furthermore, we highlight that research requires resources, including people with expertise both in technical areas and in policy translation, as well as stronger academic infrastructure and more financing—all of which are scarce in Mozambique, as in other LMICs.

In conclusion, to support the continued development of the research-to-health policy pipeline in Mozambique, we recommend the following:

- Research institutions and universities should be involved in helping to assess the impacts of community-based health projects so that the data generated from these projects are included in both evidence dissemination and policy improvement.
- Research institutions should collaborate to develop a platform to consolidate health system-related research, making evidence accessible and useful to policy makers.
- Research institutions should invest in improving knowledge translation mechanisms that support local, national, and international dissemination of research findings.
- Research and technical institutions should support opportunities for policy makers to ask questions, assess evidence, and discuss how to apply research findings to policy; and policy makers should make time for capacity development in this area.
- The government should further incentivize the conduct of HPSR by universities and professional technical institutes, creating more opportunities for students in the country to participate in and understand research and to generate more research relevant to national health policy priorities.
- The government should actively engage a wide range of institutions in consultations and dia-logues on articulating HPSR research priorities and health sector policies.
- Domestic and international donors should reinforce funding for research institutions and universities in Mozambique to enable researchers to pursue domestically relevant HPSR topics.
- National and international donors should fund more Mozambican researchers to study how research-based evidence can best be applied during health system policy design.

## Translation

**Em Português**

**Utilização da Investigação Para o Desenvolvimento e Implementação de Políticas de Sistemas de Saúde em Moçambique: Um estudo Descritivo**

**Mensagens-chave:**

- As prioridades da investigação em Moçambique nem sempre estão alinhadas as actuais reformas do sector da saúde, porque grande parte do financiamento para PPSS em Moçambique provém de doadores internacionais com agendas globais de investigação.
- Moçambique ainda não tem plataformas e mecanismos adequados e funcionais para os investigadores divulgarem resultados de pesquisas baseadas em evidências aos decisores políticos nacionais para informar questões políticas.

**Resumo**

O desenvolvimento de políticas baseadas em evidências é um desafio primordial que os países de baixa e média renda enfrentam. Moçambique tem cada vez mais Políticas geradas por evidências e Investigação de Sistemas por um lado, e por outro possui, igualmente um vasto leque de políticas, intervenções de saúde pública, tecnologias e soluções inovadoras que estão a ser implementadas na mesma linha. Opresente estudo, usou métodos qualitativos, para explorar a relação entre Pesquisas em Políticas e Sistemas de Saúde (PPSS), e o desenvolvimento e implementação de políticas baseadas em evidências em Moçambique. Os dados deste estudo foram colhidos através de uma revisão de literatura e entrevistas em profundidade com investigadores, especialistas e decisores políticos. A análise procurou avaliar os obstáculos à utilização de evidências científicas e identificar potenciais oportunidades para melhorar a utilização de evidências no desenvolvimento e implementação das políticas nacionais de saúde.

O estudo mapeou e identificou um número considerável de instituições de investigação no país, que realizam actividades na área da saúde em geral, e especificamente em sistemas de saúde, produzindo evidências científicas sólidas. Apartir deste constatou se existir também uma tendência crescente por parte dos fazedores de políticas, e decisores políticos em utilizar, os resultados da investigação durante a concepção, formulação e implementação das políticas de saúde. A maioria das PPSS que estão a ser conduzidas em Moçambique, são financiadas por doadores internacionais, e estão focadaspara questões de investigação de interesse internacional. Por isso, a investigação gerada em Moçambique nem sempre aborda questões relevantes para a agenda local de desenvolvimento do sistema de saúde.

Embora Moçambique tenha muitos pontos de produção e venda de “literatura cinzenta”, existem poucas publicações, que permitem a tradução de evidências de investigação em políticas pelo facto de uma grande parte das Evidências geradas serem divulgadas na língua inglesa, em detrimento da portuguesa, a língua oficial falada em Moçambique, e em artigos científicos de leitura complexa e não em formatos que facilitariam uma melhor consulta e utilização por parte dos decisores políticos, como por exemplo “PolicesBriefs,” constituindo assim uma barreira importante para a compreensão e utilização destas evidências a nível nacional. A principal recomendação emergente do estudo é que as instituições de investigação e decisores políticos em Moçambique devem colaborar, para desenvolver uma plataforma que permita, uma melhor consolidação dePPSS para torná-la acessível e útil aos decisores políticos.

**Peer Reviewed**
